# A mixed-methods formative process evaluation of the falls management exercise programme in an English county

**DOI:** 10.1186/s12889-025-23737-6

**Published:** 2025-08-01

**Authors:** Patricia Jessiman, Ruth Salway, Rona Campbell

**Affiliations:** Department of Population Health Sciences, Bristol Medical School, University of Bristol, Bristol, UK

## Abstract

**Background:**

The Falls Management Exercise (FaME) Programme has been shown in clinical trials to reduce falls amongst the over 65s. FaME was implemented in one large, rural county in England. The current study is a formative process evaluation measuring the impact on those completing the FaME intervention, and examines service reach, fidelity, acceptability, and maintenance of outcomes.

**Methods:**

Anonymised routine data was collected from 110 participants at baseline, the end of the programme and at three-months follow-up. Median and IQR at baseline and end of FaME for self-reported falls, fear of falling, physical activity, quality of life, loneliness, Timed-up-and-go, Turn 180^0^ and Functional Reach were calculated, and differences were compared using a Wilcoxon signed-rank test. Qualitative data were collected through interviews with intervention commissioners (*N* = 5), delivery staff (*N* = 9) and participants (*N* = 32) and their carers (*N* = 8) and eight structured observations of FaME classes, and analysed using the Framework method.

**Results:**

Of the 110 participants who started the programme and consented to routine data being collected for the study, 90% remained on programme and completed the final 24th session; 75% completed at least 75% or more of the 24-week course. Median levels of Timed-up-and-go, Functional Reach and the Turn 180^o^ test improved across the 110 participants between baseline and the end of the FaME intervention (*P* < 0.001). Fear of falling and self-reported falls decreased on average by the end of the intervention (*P* < 0.001). Participants were highly motivated to attend, with completers reporting a range of benefits attributed to the intervention.

**Discussion:**

The primary aim of reducing falls amongst the over-65s participating in FaME was met and the service was acceptable to participants. There remain concerns around service reach, and the potential to widen health inequalities. The main risk to the maintenance of the FaME intervention across the county is the recruitment and retention of programme staff.

**Conclusion:**

This study finds evidence that despite the anticipated challenges of implementation in a rural county with poor infrastructure, the intervention achieved improved outcomes for participants.

**Study registration:**

Researchregistry9560.

**Supplementary Information:**

The online version contains supplementary material available at 10.1186/s12889-025-23737-6.

## Background

Falls amongst older adults are a major cause of pain and distress, injury, loss of independence, and mortality [1]. In England, around one third of adults over 65 years fall at least once a year, rising to around half of those over 80 years [1]. In addition to age, risk factors for falls include (but are not limited to) previous experience of falls, impaired balance, loss of muscle strength, visual impairment, use of multiple medications and gait difficulty [2]. There is also evidence that older adults living in more deprived areas may be at increased risk [3, 4]. As well as the distress to individuals and their families, falls are a high cost to health and social care services [1, 2, 5]. This cost is likely to increase without significant intervention given the ageing UK population; the proportion of over 85s is projected to increase from 2.5% (1.6 million) to 3.5% (2.6 million) over the next 15 years [6].

**Fig. 1:**
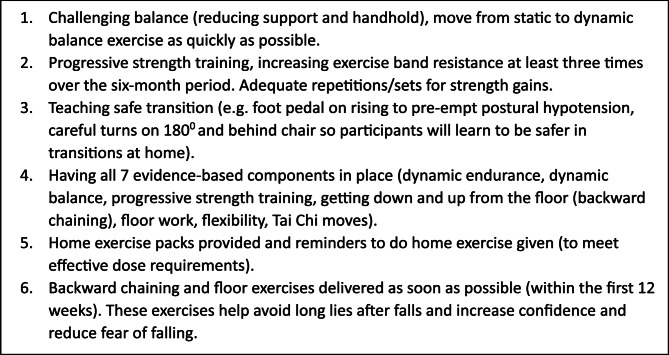
FaME essential intervention content [9, 10]

Exercise, particularly that which involves balance and functional training to increase muscle strength, has been shown to reduce falls amongst older adults [7]. The UK Chief Medical Officers’ physical activity guidelines recommend older adults undertake 150 minutes of moderate to vigorous physical activity (MVPA) per week and take part in activity promoting muscle strength and balance at least two days per week [8]. The Falls Management Exercise (FaME) Programme is a 24-week structured group exercise programme delivered by accredited Postural Stability Instructors (PSIs), and designed to improve strength, balance, flexibility and functional floor and gait skills. Participants are also encouraged to exercise at home with the intention that participants complete 50 hours’ worth of exercise over 24 weeks. Essential intervention content has been identified in previous studies of FaME [9, 10] and is summarized in Fig. 1. The programme was found to be effective in preventing falls amongst older adults in a randomised clinical trial (the ProAct65 + trial) comparing FaME with home-based exercise (the Otago Exercise Programme (OEP)) and usual care [11]. The proportion of participants in the FaME arm of the trial reporting at least 150 min of MVPA per week rose from 40% at baseline to 49% at 12 months after the intervention, compared with a rise of 41–43% in the OEP arm and 37.5–38% in the usual care arm. There was also a reduction in falls 12 months post-intervention amongst those in the FaME arm (Incidence rate ratio 0.74, 95% CI 0.55 to 0.99, *P* = 0.042) compared to usual care [11]. A smaller study of FaME in real world, routinely commissioned programmes in England found significant improvements in confidence in balance, fear of falling, functional reach and Timed-up-and-go tests (all *P* < 0.001) and the Turn 180^o^ test (*P* = 0.008) though these were not maintained at 6 months [12]. Participant attrition in this study was high, with 143 of 348 participants (41%) attending 75% of classes. Falls incidence was lower at the end of the programme and at 6 months later, but the differences were not significant [12]. Maintenance of at least 150 min per week of MVPA after the FaME intervention is finished is also associated with a reduction in falls [11, 13]. Strength and balance programmes are recommended as a falls prevention measure by the National Institute for Health and Care Excellence [14] and in the National Falls Prevention Coordination Group Falls and Fractures Consensus statement [15]. FaME was also recognised by Public Health England (PHE) as a cost-effective approach to preventing falls amongst community-dwelling older adults [16]. As a consequence, local authority public health teams across England are increasingly commissioning the FaME programme as a falls prevention measure for their local population of older adults.

FaME was commissioned by the local authority in one large, rural county in England (Lincolnshire) with an ageing and largely White population (UK Census data 2021 indicated 23.9% aged 65 years and over; 96% White [17]) and no pre-existing strength and balance services for older adults. The implementation of the FaME programme was expected to be especially challenging in a county with poor transport infrastructure, rural and coastal geographies, and pockets of deprivation [18]. The target population were those aged 65 and over living in the county deemed to be at heightened risk of falling, with the ambition that over 300 participants would complete FaME between May 2023 and June 2024. Classes were rolled out to cohorts of up to 14 participants who started and ended the 24-week programme at the same time (rather than as a rolling programme [19]. Participants were encouraged to stay for a half-hour social session after each class to encourage group bonding and help address concerns about social isolation and loneliness amongst older adults following the Covid19 pandemic. A logic model for the FaME intervention [20] was adapted for the intervention following consultation with local authority staff, the service manager, and two public representatives and is available in Supplementary File 1.

The local authority applied to the National Institute for Health Research (NIHR) Public Health Intervention Responsive Studies Teams (PHIRST) scheme for evaluative support for the FaME rollout to help determine whether good outcomes (reducing falls incidence and fear of falling, improving functional mobility and physical activity levels) could be achieved and maintained, and whether FaME was both accessible and acceptable to the intended target population of over-65s at risk of falls. The aim of the current study was therefore to conduct a formative process evaluation to measure the impact on those completing the FaME intervention, and examine service reach, fidelity, acceptability, and maintenance of outcomes.

## Methods

The study was sponsored by the University of Bristol and received ethical approval from the NHS Health Research Authority (ref 23/LO/0554).

### Study design

The study was a mixed-method formative service evaluation across multiple delivery sites. Routine data were collected by the delivery organisation at baseline, end of the 24-week programme, and three months after the end of the programme. Qualitative data were collected through interviews with intervention commissioners, delivery staff and participants and structured observations of FaME classes. The study period was May 2023 until June 2024, with follow up data collected up until October 2024.

### Service referral pathways

The local authority commissioning team set out the criteria for referral onto the FaME service, including that referrals should only be accepted from health and care professionals including but not limited to general practitioners (GPs), nurses, physiotherapists, and occupational therapists. Preventing participants self-referring straight onto the service was intended to avoid using limited service capacity for the ‘worried well’ or those residents who were more able to find out and apply independently, and hence run the risk of widening health inequalities. There had been an ambition to use Population Health Management (PHM) data to identify a cohort of at-risk older adults using proxy measures of falls risk (e.g. hospitalisation in the previous 12 months; frailty indicators) and share this with health care providers to encourage referrals but this was delayed and did not happen during the study period. The delivery organisation promoted the service widely to health professionals across the county, building on their existing relationships with them. The referral rate increased quickly as a result, and many potential participants were placed on a waiting list, so the local authority did not pursue the use of PHM data further.

### Routine data

Consent for anonymised routine data to be shared with the research team was sought from participants starting FaME between May 2023 and March 2024 to ensure that outcome data at the end of the intervention would be available by the end of the study period (October 2024). This included demographic data on age and gender; we did not request consent for data on ethnicity and postcodes to be shared with the research team. Data collected at baseline and at the end of the 24-week programme included self-reported clinical outcome measures: fear of falling Short Falls Efficacy Scale (FES-1) [21], quality of life Eq. 5D5L [22], the UCLA Loneliness scale [23], self-reported number of falls in the previous three months, and self-reported physical activity level (minutes per week). All of these measures were collected by staff from the delivery organisation either during a face to face visit or telephone call with participants. For the measure of physical activity, participants were asked the question ‘*how many minutes of moderate physical activity do you do per week?*’ and ‘moderate’ defined as ‘*when you feel more out of breath and your heart is beating faster’*.

Functional measures of balance, mobility and falls risk were measured using Timed-up-and-go [24], Turn 180^0^ [25] and Functional Reach [26] tests. Each of these three functional measures were collected by PSIs using an ordinal scale 1–6 supplied by the FaME training provider (see Supplementary File 2 for full details), which was treated as continuous in the analysis. Data collected at 3 months follow-up included self-reported falls in the previous three months, fear of falling FES-1, physical activity level, and quality of life.

Demographic data (age, gender) were collected at baseline, and attendance recorded throughout the 24 weeks. Data on referral rates and those declining the opportunity to participate in FaME were also collected.

Descriptive summaries of demographics and self-reported falls were reported at baseline. Median and IQR at baseline and end of FaME for self-reported falls, FES-1, minutes of physical activity per week, Eq. 5DSL, UCLA loneliness, Timed-up-and-go, Turn 180^0^ and Functional Reach were calculated, and differences were compared using a Wilcoxon signed-rank test. Similarly, differences in self-reported falls, FES-1, minutes of physical activity per week and Eq. 5DSL were compared between baseline and 3-months post-intervention. Change in measures between time points were classified as ‘worsen’, ‘improve’ or ‘no change’, using thresholds of 4 or more for FES-1 (a scale of 16–64), a change of 30 min or more in weekly physical activity, and Eq. 5DSL thresholds based on baseline standard deviation (SD), with 0.5-1 SD classed as a small change in Eq. 5DSL, and 1SD or more as a large change [27]. Data were analysed in Stata v18.0 [28].

### Qualitative data collection

Two rounds of qualitative interviews were undertaken with professionals towards the start of the implementation period and again towards the end. These included staff from the local authority public health team, the service manager and PSIs. The service manager and local authority staff were known to the research team through their involvement in setting up the study; PSIs were recruited via an email forwarded by the service manager. The majority of interviews with professionals were face to face with a small number undertaken online or by telephone. Topic guides for the interviews were developed for the study (Supplementary Files 3 and 4).

The study also aimed to recruit health professionals throughout the county who were referring patients to the FaME programme. An invitation to complete a short online survey was sent to health professionals through inclusion in a regular newsletter from the delivery provider, with an option to volunteer for a qualitative telephone interview.

Older adults eligible and referred to FaME were interviewed for the study between October 2023 and June 2024. The sample included participants mid-way through the 24-week intervention (‘current attenders’), and others at least four weeks after they had completed it and had attended at least 75% of classes (‘completers’). All of these participants were recruited by researchers who attended a FaME class to observe the session and share information about the study. These participants were interviewed face to face, usually in their own homes. Where possible we also spoke with carers/spouses of FaME participants if they were present during the interview, to understand their perspective on the FaME intervention and its impact on participants, and any impact on spouses/carers themselves. The sample also included telephone interviews with participants who had dropped out of the intervention mid-way through, and those who had been referred but declined to attend. These participants were recruited with the help of the delivery organisation, who asked for consent for the research team to contact them.

In all cases those who participated in qualitative interviews were provided with an information sheet about the study and provided written consent to interview participation. Those older adults referred to FaME and their spouses/carers received a £30 high street voucher for their participation. A topic guide for these interviews was developed for the study (Supplementary File 5).

Qualitative interviews were recorded and transcribed verbatim and analysed using the Framework method, a thematic analysis approach frequently used in health service evaluation [29]. The lead researcher developed a draft analytical framework that included the key themes identified in the data and relevant to the research questions. This analytical framework was reviewed by other members of the research team and the two public members of the study management group, and revised until agreement was reached. A systematic approach to data management was adopted, with the lead researcher coding the transcripts into the analytical framework using NVivo software [30]. The analysis continued using Framework matrices as a detailed and accessible overview of the data populating each theme from every data collection event.

### Structured observations of fame classes

An observation framework was developed for FaME classes to identify how FaME was being delivered, determine where the essential intervention content was being delivered (see Fig. 1) and capture details of the physical environment and the nature of interactions between participants and instructors. Consent was sought from participants one week in advance of observations.

### Data triangulation

Qualitative and quantitative data were collected and analysed separately, as they addressed separate research questions. The quantitative data was used to answer questions around impact and service reach, while qualitative data largely examined fidelity, acceptability and outcome maintenance. However where the two datasets overlap a research question, for example impact on clinical and functional outcomes and participants’ perceptions of impact, these are presented side by side in the results section.

## Results

### Routine data sample size and participant characteristics

Of the 145 participants who commenced FaME classes across the county between May 2023 and March 2024, 110 (76%) consented to routine data being shared with the study team. 65% were female and the mean age was 79.5 years. Over half had experienced at least one fall in the previous three months prior to starting FaME (see Table 1). Three-month follow-up data were available for 63 (57%) participants, and so caution must be applied when interpreting follow-up data.

**Table 1:** Profile of routine data participants at baseline

	*n*	%
All		
Gender		
* Male*	39	35%
* Female*	71	65%
* Age*		
* 65–74 years*	26	24%
* 75–79 years*	30	27%
* 80–84 years*	28	25%
* 85 + years*	26	24%
* Number of falls in previous 3 months at baseline*		
0	42	43%
* 1*	37	38%
* 2*	14	14%
* 3*	5	5%
* Missing*	12	

### Qualitative interview sample

In total seven interviews were conducted with commissioners and the service manager, lasting between 45 and 105 min. Five of these were conducted with staff from the local authority public health team (one participant was interviewed at both timepoints), and two interviews were conducted with the service manager for FaME (quotes from this group are identified below as ‘CM’). Eight interviews were conducted with PSIs (three PSIs were interviewed at both timepoints) and these interviews lasted between 35 and 65 min.

Thirty-two older adults eligible and referred to the FaME service were interviewed. Of these, 19 were female and 13 male, aged between 67 and 98 years. The sample included 10 current attenders, and 17 completers. A further five were interviewed by telephone, three of whom had stopped attending FaME before the 24 weeks were completed, and two who had been referred but declined to attend. Quotes from participants are identified below as ‘P’. Eight spouses of current attenders/completers were also interviewed (four male, four female). These interviews lasted between 30 and 79 min.

Despite repeated invitations to complete a short online survey we were unable to recruit any referring health professionals to the study.

### Referrals to the service

Routine data collected during the study period indicates that 62% of participants were referred by a GP.

Most FaME participants interviewed for the study knew they had been referred to the intervention by a health professional, usually a GP or physiotherapist. Few recalled being given detailed information about the service other than it was ‘for strength and balance’ or ‘to prevent falls’. Some were unaware that they had been referred until they were called by the delivery organisation offering a place. The quote below is from a participant who went on to complete FaME but was not aware how or why she was referred.

*I didn’t know about it. I suddenly received this letter*,* saying to go to this keep fit meeting*,* and I thought*,* “Well*,* I haven’t said I would do this.” It was from my doctor’s surgery*,* so I don’t know. Somebody must have put my name down.*

P17, Female

### PSI recruitment and training

At the start of the study period, the delivery provider had two accredited PSIs available to deliver FaME and had to recruit and organise training for more instructors to deliver the service across the county. Efforts were made to recruit instructors who had experience in delivering exercise classes to older adults. PSI training was delivered by an external training company through a blend of face-to-face training days and online learning over 16 weeks. The number of PSIs increased throughout the pilot to up to seven PSIs (a mix of employed and freelance staff) delivering the service across the county at any one time. Freelance staff were offered the training free of charge if they committed to delivering at least two 24-week FaME courses. PSIs reported finding the training course intensive and time consuming, and fitting PSI training around their existing workload was often tricky for freelance staff. However they also report that the training informed their wider practice.

*It has informed my classes as well*,* my existing classes*,* very much so actually. I already did a seated class*,* as a freelance*,* for a local village. I didn’t really have much experience of seated classes because mine are all standing*,* so it definitely helped with the writing of the seated classes every week.*

PSI4

There was some turnover of PSIs throughout the pilot, with freelance instructors more vulnerable to leaving the service.

*I think that we would be able to keep more staff if we had them as part of [our organisation]. There are things that as a member of staff you get whereas as a freelancer you don’t. …Again*,* I can’t put myself in their shoes*,* but I can imagine it’s lonely*,* but they’re just going to do delivery and then we try and meet up every couple of weeks*,* but everyone’s times are so different and it’s really hard to keep that team together.*

CM1

Travel time may also have impacted PSI retention, with instructors often having over an hour’s travel to get to class locations throughout the county. This also limited capacity to increase the number of classes delivered at any one time.

### Number and distribution of fame classes across the county

Twenty-four FaME courses, reaching 287 participants, started across the county in community venues during the study period. There was a good geographical spread across the seven districts of the county, including rural and coastal areas. There was a three-month period (January-March 2024) during which no new FaME classes were started in the county while contacting arrangements between the delivery provider and the local authority were revised in order to extend the original service agreement for a further 12 months. This interruption caused an increase in the number of participants on the waiting list and some uncertainty over the security of the service for both PSIs and health professionals referring patients to FaME. There were also limitations around the delivery provider’s capacity to offer immediate entry to a local class for new referrals, because of the limited number of trained PSIs, travel time across the county, and the need to have enough referrals within a locality to justify starting a new class. This meant that some referrals were placed on a waiting list for several months before starting the FaME programme, which may have affected health professionals’ willingness to refer patients.

*[Health professionals] are hearing from those people who had positive experiences about FaME and want to get their patients involved in it. I’ve had the odd finger wagging experience around it was all well and good you’ve setting these things up and then saying they’re available to people and then they’re not really available to people are they.*

CM5

The delivery provider used demand for the service, measured by interest from health professionals and geographical location of those participants referred and on the waiting list, to determine where in the county to locate FaME classes. There was no evidence, either from interviews with staff or analysis of class location, that classes were being preferentially sited in areas with higher socio-economic deprivation.

*It’s a mixture between the wait list size*,* how big*,* how many people we’ve got coming in for that specific area. But also if a referrer comes to us and says we’ve got this many people*,* we need a group going on*,* can you do it? We will then do that.*

CM1

### Completion and attrition rates

Of the 110 participants who started the programme and consented to routine data being collected for the study, 99 (90%) remained on programme and completed the final 24th session; 83 (75%) completed at least 75% or more of the 24-week course. Key drop out points were around halfway (sessions 9–14) and towards the end (sessions 20–24). There were no differences in completion rates or number of sessions by age or gender. PSIs were aware of high rates of attrition in some FaME classes and attributed this to ill health of self or a family member, and problems with transport.

*Two that started and then couldn’t make it was because their husbands became ill and were then in hospital …[]… therefore they didn’t have the transport in or the support to be able to get to the lessons.*

PSI6

Only 10 (9%) of participants completed all 24 classes. PSIs reported that the most common reasons given by participants for missing classes (but continuing to attend later classes) were ill health or planned holidays. This is supported by data from the interviews with FaME attendees, most of whom reported missing classes at some point throughout the 24- week period. Frequent reasons given include holidays, health appointments, ill health (of self or spouse), injury (caused by a fall), or lack of transport. Most interviewed were highly motivated to attend and make the most of the service, and were concerned about the cost implications of those participants who dropped out or failed to attend so regularly.

*Now*,* it would take a tremendous lot to stop me going. I’d have to be ill or some major thing to stop me from going because I’m very keen on it.*

P1, Male

Transportation is problematic in many areas of the county for participants who do not drive, particularly in rural areas, although even in urban areas participants with reduced mobility may be unable to access public transport. PSIs were aware of this, and keen to site classes where they were most accessible, however the wide and often rural geographical area that participants were drawn from means this was not always possible.

*The transport problem…[]…especially here*,* many of the people*,* many of the participants did not drive and they relied on either public transport*,* i.e. buses*,* or taxis to get them to the classes and that proves to be unreliable and expensive. That seemed to be a major issue and it does seem to be continuing to be a major issue for people.*

PSI5

### Decline of service

Routine quantitative data collected by the delivery organisation shows that 114 participants referred to the service by a health professional during the study period declined to attend. Reasons for decline were recorded (see Table 2 below). Where known, the most common reason was not wanting this type of support (*N* = 42). The limited qualitative data from two participants interviewed who declined to attend suggests that participants did not know not very much about the service on referral.

*[The consultant] was going to refer me to a falls clinic. But I had a different impression of what a falls clinic would be to what I actually discovered*,* when I was contacted… Yes. I thought I was going to something like physio on a one-to-one basis.*

P15 Female

*I’m housebound. I’m on the way out….I live a normal life*,* except I can’t walk…[] It’s in [village] and I live in [Town]*,* I can’t get there.*

P31 Female

This suggests that health professionals may not have been providing adequate information about the FaME service or enquiring whether participants would like this sort of support before referral. We do not know from these data whether participants would have preferred a different type of falls prevention service, or no support at all. Health conditions were also a common reason for decline (*N* = 22), and not being able to travel to the FaME class (*N* = 18).

**Table 2:** Reasons for decline of service

Reason for decline of service	*N* = 114
Do not want support	42
Health conditions	22
Travel barrier	18
Physical injury (includes broken bones, recent surgery)	6
Not the right time	3
Housebound	1
Mental Health	1
Unable to contact/Unknown	21

### Delivery of fame classes

Eight FaME classes were observed across eight different locations/groups and five PSIs. The earliest week observed was week five of the 24-week course, and the latest week 23. Classes followed a similar format, moving through warm-up and stretch exercises, then aerobic, balance, and resistance training, ending with a cooldown and stretch. Floor work (backward chaining and floor-based exercises) was observed on three occasions, and this was the section of the class where there was most variation across participants in what they were able to do. Not all participants attempted floor work, and were given alternative, seated exercises. In several cases the class ran slightly longer than an hour, particularly when floor work was part of the session, but none of the participants were concerned about this. Reminders to exercise at home between classes were seen in only four of the eight observations, although it is possible that some participants were spoken to about this individually during the social session. Observation of eight classes and participant interviews indicated that the six key elements of FaME outlined in Fig. 1 were being delivered. PSIs interviewed for the study were highly aware of the need to maintain programme fidelity.

*Obviously in the training they hammer home the importance of fidelity*,* because if we don’t have the fidelity it’s not going to work. Yes*,* initially when you start delivering I think it’s kind of a little bit*,* “Oh my goodness. Am I getting everything right?” But you soon fall into a groove with it*.

PSI2

In all observations, class participants stayed for a shared social time at the end of the class. This, and the group dynamic, was valued by the majority of participants in the qualitative sample. Most believed that their motivation to exercise was helped by being in a group class, and much stronger than if they were exercising alone. Participants were giving and receiving encouragement during the more difficult elements. Some reported making new friends, with plans to keep in touch after completion of the course.

*They were all friendly. And it was good*,* because they helped you*,* as well …. It was just so nice. Everybody was. Because you did all the exercises*,* and then you sat down and talked to everybody. It was really nice.*

P18 Male

### Functional, clinical and quality of life outcomes

Median levels of functional measures (Timed-up-and-go, Functional Reach and the Turn 180^o^ test) improved across the 110 participants between baseline and the end of the FaME intervention (Table 3). 77% of participants improved their Timed-up-and-go score between baseline and end-FaME; 63% improved their Functional Reach, and 59% their Turn 180^0^ score (Table 4).

Scores on the FES-1 Scale decreased on average by the end of the intervention, and remained lower than baseline at 3-months follow up (note the low sample size at 3-month follow up). The number of self-reported falls in the previous three months was lower on average at the end of the 24-week FaME programme than at baseline and remained lower at follow-up. At the end of the intervention, 53% of participants reported fewer falls than at baseline (44% no change) and this was maintained at 3-month follow-up (53%).

Self-reported quality of life (Eq. 5D5) was on average higher at the end of the intervention, and remained so at follow-up. 95% of participants showed at least a small increase at the end of the FaME intervention (between 0.5 and 1 SD) and 95% at follow-up. There was a larger increase in quality of life (greater than 1 SD) for 26% of participants at the end of FaME and 17% at follow-up. There was some reduction in self-reported loneliness on average, though 63% of participants report no change between baseline and the end of FaME (data are missing for 35% of participants).

**Table 3:** Median outcome measures at baseline, end-FaME and 3 months post-intervention

	Baseline		End of FaME		3 month follow-up^1^		Baseline vs. end of FaME	Baseline vs. 3 month^1^
	Median	IQR	Median	IQR	Median	IQR	*p*-value^2^	*p*-value^2^
Number of falls	1	1	0	0	0	0	< 0.001	< 0.001
Short falls efficacy scale	13	4	9	3	9	3	< 0.001	< 0.001
Physical activity	0	20	70	30	70	40	< 0.001	< 0.001
Equation 5D5L	0.66	0.22	0.74	0.20	0.74	0.22	< 0.001	< 0.001
UCLA loneliness scale	3	1	3	1			0.001	
Timed up and^3^ go	3	1	2	1			< 0.001	
Functional reach^3^	3	1	2	1.5			< 0.001	
Turn 180^° 3^	2	2	1	0.5			< 0.001	

^1^Note smaller sample size for 3-month follow-up *N* = 63

^2^Wilcoxon signed-rank test

^3^Collected using an ordinal scale 1–6 (see Supplementary File 2 for), which was treated as continuous in the analysis

**Table 4:** Proportion of participants who got worse, experienced no change or improved on outcome measures

	Worsen		No change		Improve	
	N	%	N	%	N	%
Between baseline and at end of FaME						
Number of falls	3	3%	42	44%	50	53%
Short falls efficacy scale^a^	4	4%	49	48%	50	49%
Physical Activity^b^	1	1%	7	7%	89	92%
Equation 5D5L: small change^c^	5	5%	0	0%	98	95%
Equation 5D5L: large change^c^	1	1%	75	73%	27	26%
UCLA loneliness scale^d^	5	7%	45	63%	22	31%
Timed up and go^e^	2	2%	21	21%	77	77%
Functional reach^e^	9	9%	28	28%	63	63%
Turn 180^o e^	3	3%	38	38%	59	59%
Between baseline and 3-months follow-up						
Number of falls	2	3%	25	43%	31	53%
Short falls efficacy scale^a^	3	5%	34	54%	26	41%
Physical activity^b^	0	0%	8	13%	52	87%
Equation 5D5L: small change^c^	3	5%	0	0%	60	95%
Equation 5D5L: large change^c^	2	3%	50	79%	11	17%

^a^Changes in FES scale (16–64) of 4 or more

^b^Changes in Physical Activity of 30 min or more

^c^Changes Eq. 5DL calculated in terms of baseline SD = 0.18 [27]:

No change: < 0.5 SD

Small change 0.5-1SD

Large change > 1 SD

^d^Data are missing for 35% of participants

^e^Collected using an ordinal scale 1–6 (see Supplementary File 2 for), which was treated as continuous in the analysis

#### Perceived impact on falls risk and quality of life

Most FaME participants interviewed for the study perceived a range of benefits they attributed to the intervention. Increased strength and stamina and improved stability were frequently reported.

*I couldn’t get in and out of the bath before…[]… I’ve been getting up and down better. I can’t put my left knee to the floor at the moment. I can my right. And I managed the other day….[]…I had the phone there in case I had to ring my husband downstairs. But I managed*,* and I did it on my own. Because I feel strengthened and*,* I don’t know*,* a bit more confident maybe.*

P4 Female

Few participants believed that attending the class would prevent future falls, because they had fallen so often they believed nothing could help.

*These days*,* I’m just terrified*,* absolutely terrified of falling over.*

*Interviewer: that hasn’t changed since the course?*

*No. I don’t think it ever will now*,* because I’ve had those very bad falls.*

P13 Male

However, most did perceive that they had learned good tips about preventing falls, particularly around posture, lifting their feet, and ‘stepping out’ when they needed to.

*I have been worrying about [falling] but because I’ve had a few months*,* now*,* without I’m getting more confident now. I think that’s got something to do with the course. Standing with your feet apart for balance*,* because normally I would stand with my legs together. I’ve got it written down actually*,* (Laughter) so I don’t forget anything. Oh*,* and lifting my feet up when I’m walking to stop tripping.*

P24 Female

Many participants interviewed for the study also reported some social benefits of FaME, particularly feeling more confident in their strength and stamina leading to them being more willing to leave the house. Some were motivated to attend other fitness classes. Those participants who reported feeling socially isolated, either through illness, or living with illness (or both), believed that spending the 24-weeks with a group of similar participants had helped their mental wellbeing.

*I’m getting to the point where I don’t even go shopping now. I feel a little bit depressed by it*,* and that’s where the class is helpful in getting me out. It has side issues and benefits that you wouldn’t perhaps think of straightaway.*

P7 Male

### Changes in physical activity level

Median levels of self-reported minutes of physical activity per week increased by the end of intervention. At baseline, 78% of participants were doing less than 30 min activity per week; by the end of FaME 56% were achieving 60–89 min, and 50% achieving 60–89 min at 3-months follow-up (Table 5). Because participants were asked to self-report physical activity levels at the end of the 24-week programme and not specifically directed to exclude it, attendance of the 60-minute FaME class may have been included by some participants at this time point. 87% of participants improved their physical activity levels between baseline and at 3 months follow-up, when they were no longer attending FaME. However, very few participants at any timepoint met the recommended 150 min of moderate to vigorous physical activity per week [30] (none at baseline, 8 (8%) at the end of the FaME intervention, and 5 (8%) at 3-months post-intervention).

**Table 5:** Level of Physical Activity at baseline, end-FaME and 3 month follow-up

	Baseline		End of FaME		3 month follow-up	
	N	%	N	%	N	%
Physical activity (mins)						
< 30 min	78	78%	1	1%	5	8%
30 −59 min	16	16%	13	13%	6	10%
60-89 min	4	4%	57	56%	31	50%
90+min	2	2%	30	30%	20	32%

### Perceived maintenance of physical activity

Several FaME participants reported that they would like to continue some form of group exercise after they had completed the 24-week FaME intervention, but were unsure anything suitable would be available. Just over half (51%) of participants who finished FaME during the study period (i.e. attended the final 24th class, though they may have missed classes during the course) were referred on to a follow-up 12-week exercise programme offered by the same delivery organisation, though in a different venue. Many respondents were reluctant to travel far to attend a new class, and almost none were keen to try online exercise classes.

*I’ve tried [online]*,* and I can’t keep the enthusiasm up very well. I’ve got to force myself to get a slot and be there and switch on at the right time*,* and this and that. I haven’t got on too well with that*,* but that’s just me.*

P7 Male

Most FaME attendees were also keen to carry on exercising with the same group of participants, both to support their confidence in attending a new class but also to maintain the social contacts they developed during FaME.

*No*,* that’s what I’m sad- Well I say sad. I don’t know. This is what I wonder. There won’t be anything to carry on*,* will there? I do hope so. It would be nice to keep the friendship of the people because I’m sure*,* when you do live on your own*,* it’s nice just to have a chat isn’t it?*

P10 Female

A small number of FaME completers had found local exercise classes aimed at older adults, and planned to attend regularly.

*I said*,* “Yes*,* I’ll try anything. I don’t mind.” And*,* yeah*,* I was pleased*,* and I liked going there. The thing that it has done for me*,* I’ve been a bit up and down since then*,* but it doesn’t matter about that*,* but it’s encouraged me to go to exercise classes. And*,* I have been to one and then I found out only this week there’s another one just up the road here somewhere. You know*,* if you don’t use it*,* you lose it.*

P16 Female

There was recognition amongst both commissioning and delivery staff that the availability of exercise programmes that would promote strength and balance in older adults across the county was poor, particularly in more rural areas.

*One of the issues we have is a strategic commissioning one*,* we don’t have anything else commissioned for people to move on to*. *There are lots of leisure and fitness programmes*,* lots of walking groups but these are not always the best for a cohort with a fear of falling*…[]…*The well-being programme might help someone find an activity that’s already going on somewhere*,* but if you haven’t got a lot of activities going on that are centred on this kind of need*,* then there won’t be anything for them to find.*

CM5

## Discussion

The current study aimed to determine whether good outcomes could be achieved and maintained for older adults at risk of falls through the implementation of FaME throughout an English county with challenges of poor infrastructure, rural and coastal geographies, and pockets of deprivation. The primary aim of implementing FaME across the county was reducing falls amongst the over-65s. For those completing FaME in this study, the number of self-reported falls in the previous three months is lower on average at the end of FaME than at baseline and remains lower at three-month follow-up. In addition, all three functional measures (timed-up-and-go, Functional Reach and the Turn 180^o^ test) improved on average across the 110 participants between baseline and the end of the FaME intervention (these measures were recorded by PSIs using an ordinal scale, which is a limitation of the study methodology). There is similar improvement in the self-reported clinical outcome measures. Scores on the Short Falls Efficacy Scale (a measure of fear of falling) improved on average by the end of the intervention and remain lower than baseline at 3-months follow up. Self-reported quality of life improved on average post-intervention, and at follow-up. These short-term outcomes for FaME in Lincolnshire are encouraging and strongly suggest that the intervention is reducing falls and fear of falling by the end of the 6-month intervention period. The short follow-up period (three months) and limited availability of data suggests caution should be applied when evaluating the longer-term impact of the intervention.

The study also sought to learn lessons from the implementation, including around service reach, fidelity, acceptability, and maintenance of outcomes, which may be applicable to other similar localities. FaME was commissioned by the local authority because it is an evidenced-based programme [11, 13, 32], and the preferred choice over other programmes (e.g. the Otago home exercise programme) because of its group delivery mode which it was hoped would address the increased risks of social isolation and loneliness post-COVID19. On average, there is some reduction between baseline and the end of FaME in self-reported loneliness, though the majority of participants show no change. Qualitative evidence suggests that participants enjoyed the company and support of others in the class. This has also been reported in a previous qualitative study of FaME, with participants strongly preferring group classes to exercise at home or alone [32].

Local authority public health staff aimed for 300 participants to complete the intervention in the first year of implementation. The achieved number fell just short of this ambition; 287 participants started FaME in the county between May 2023 and June 2024, though many of these would not complete the 6-month intervention until up to six months later. This was in part due to the time taken to recruit and train enough PSIs to deliver the intervention, and the 3-month service interruption during which no new classes could start while contracting arrangements were reviewed. Those attending FaME in the county had a mean age of 80 years at the start of the intervention, over half had experienced at least one fall in the previous three months and had a high concern about falling, and most were referred by a health professional, suggesting that the referral criteria were largely being met. It is a limitation of the current study that we do not have information on the ethnicity or deprivation-status of participants. Despite restricting referrals to health professionals it is still possible that service implementation was demand- rather than needs-driven. This is evidenced by the absence of any strategic approach to locating FaME classes in area of higher economic deprivation, and feedback from health professionals to commissioning staff that the service was unavailable in their area. Implementing the planned use of Population Health Management (PHM) data to identify residents of Lincolnshire most in need (using indicators of falls risk including frailty and hospitalisation) would address the risk of widening health inequalities. It would also offer the potential to evaluate the longer term health and social care outcomes of those attending FaME. It is a limitation of the current study that we were unable to recruit referring health professionals to the study and we have limited understanding of their awareness of the FaME programme and motivation to refer patients.

In the current study 90% of participants starting FaME classes remained in the programme and completed the final 24th session, with 83 of the 110 participants (75%) completing at least 75% or more of the 24-week course. This is higher than in a previous evaluation of FaME in a real world setting in which 143 of 348 starters (41%) completed at least 75% of classes [12]. In other parts of England FaME is being delivered either as a rolling programme where participants can join or leave at any time, or as a shorter course [19]. Delivering in cohorts and for a full 24 weeks may help build social bonds between participants and aid retention, as well as promote a larger dose of the intervention. The challenges of poor transport and rural infrastructure, and paucity of suitable alternative exercise provision in the county may also have introduced an element of bias here, with those agreeing to start the programme needing to carefully consider the time and resources needed to attend and therefore possibly being more committed. Taken together with qualitative data suggesting participants were highly motivated to attend, and completers reporting a range of benefits attributed to the intervention including increased strength, stamina and social benefits, this strongly indicates that the intervention was acceptable to those who participated. Similar benefits were also described in a previous qualitative study of FaME participants [33]. However, there was also a high number of eligible participants who declined to attend, often because they did not want this type of support. Poor health, lack of transportation and dislike of group classes with a social element were also cited as reasons for declining the service. Service reach would likely be improved by emphasising that the social element is voluntary and improving transport options for potential participants.

FaME is a structured programme and the fidelity of implementation in Lincolnshire is considered with reference to the essential intervention content (Fig. 1) and any adaptations to the programme or differences across classes or individual instructors. Observation of eight classes and participant interviews indicated that the six key elements of FaME were being delivered. PSIs report being highly conscious of programme fidelity. There was inconsistency in the reminders given by PSIs to exercise at home between classes but otherwise there was little variation across instructors in how the programme was delivered. This study did not measure participants’ adherence to exercising at home between classes and therefore cannot comment on the proportion that completed the recommended 50 hours’ worth of exercise over 24 weeks.

The main risk to the maintenance of the FaME intervention across the county is the recruitment and retention of sufficient PSIs to deliver the service, a challenge also faced by other local authorities implementing FaME [34]. PSIs found the 16-week training course challenging to fit around existing commitments, although most who completed it report it had improved their practice. There remain challenges for the delivery provider with staff retention. Commissioners and managers interviewed for the study consider freelance staff more vulnerable to leaving the service than those directly employed by the service provider. Training PSIs is expensive and time consuming. Careful management of PSI retention will continue to be important to the maintenance of the service and ensuring availability of FaME classes across the county.

On a participant level, maintenance of self-reported physical activity (important for falls-prevention) in the short-term was high, although data are very limited. Self-reported minutes of physical activity per week improved on average by the end of the intervention, and this was maintained at three-months follow-up. However, very few participants at any timepoint met the recommended 150 min of moderate to vigorous physical activity per week [30]. A systematic review of physical activity amongst older adults identified distance, lack of facilities, poor transport and cost amongst the key barriers [35]. There was recognition amongst all participants of the lack of exercise programmes suitable for older adults across the county, particularly in more rural areas, and efforts to both increase the availability and accessibility of these will need to be continued.

## Conclusion

This mixed-methods process evaluation of FaME shows evidence that despite the anticipated challenges of implementation in a rural county with poor infrastructure, rural and coastal geographies and pockets of deprivation, the intervention achieved improved outcomes for participants. These include a reduction in falls incidence and the fear of falling, improved physical activity levels and improved quality of life between baseline and the end of the intervention. These were maintained at 3-months follow up, though the sample size at this stage is small and results should be treated with caution. Service acceptability was good, with a high proportion of participants completing the 24-week course and reporting a range of benefits to everyday life. Service reach was challenged by the absence of any strategic approach to locating FaME classes in areas of high-need, and poor transport infrastructure in the county. The availability, acceptability and accessibility of activities that will continue to promote muscle strength and balance for participants who have completed FaME is low across the county, and efforts to improve this will be necessary to maintain the positive effects of the FaME intervention.

## Supplementary Information

Supplementary Material 1.

Supplementary Material 2.

Supplementary Material 3.

Supplementary Material 4.

Supplementary Material 5.

## Data Availability

Anonymised qualitative transcripts used during the current study are available in the data.bris repository 10.5523/bris.kjwkmo6jyf9s2a6yo813f1kq6.

## Abbreviations

FaME: Falls Management Exercise (FaME) Programme
MVPA: Moderate to vigorous physical activity
NIHR: National Institute for Health Research
PHIRST: Public Health Intervention Responsive Studies Teams
PHE: Public Health England
PSI: Postural Stability Instructor
