# Supplementary material for: A mixed-methods formative process evaluation of the falls management exercise programme in an English county
Source: BMC Public Health. 2025 Aug 1;25:2609. doi: 10.1186/s12889-025-23737-6 (PMC12315209; doi:10.1186/s12889-025-23737-6)
Supplement: Supplementary file 1 — Supplementary Material 1. [file 12889_2025_23737_MOESM1_ESM.docx]

Funding (Covid reserves)

**Infrastructure:**

Postural Stability Instructor (PSI) staff

Venues

Equipment

Digital offer - Gloji gym

Printed resources.

FaME commissioning toolkit

Delivery co-ordination: One You Lincolnshire, Integrated Lifestyle Service

Population Health Management Dataset (PHM)

Engagement with Primary care Networks (PCN)

Service Specification Developed

Recruitment of level 3 Instructors

**Training:**

Postural Stability Instructors (PSI)

Behaviour Change

Make Every Contact Count (MECC)

Caseload management system

Identification of cohorts via PHM

**FaME delivered:**

Courses scheduled.

Identification and enrolment of older people onto courses

Assessments undertaken.

FaME course delivery

Upskilled workforce

People have attended 6-month FaME programmes.

Assessments and data collection at baseline, completion, and 3 months after the completion

Follow up for people who not completed done and recorded.

Participants signposted to after programme activities (including Move more pathway at OYL)

Recruitment and training results in an increased pool of PSIs

People receive up to full 6 month ‘dose’ of classes.

Classes are progressive and tailored, following the FaME programme.

Group sessions are social and people bond as a group.

Physical activity improves physical and mental wellbeing.

Classes delivered across the county proportionally to population needs.

Supportive relationship PSI/participants

**Less use of healthcare:**

Fewer falls

Fewer emergency hospital admissions

Better long-term condition management

**Less use of social care:**

Continued independence.

**Unintended effects:**

Increased risk-taking.

**Inputs**

**Activities**

**Outputs**

**Causal Mechanisms**

**Short Term Benefits**

**Long Term Benefits**

**Health benefits:**

People become more physically active.

Strength and balance improve.

Fear of falling decreases

Confidence in daily activities improves.

Carer/family Benefits.

**Wider benefits:**

People less socially isolated

Increased awareness of local 'offer' - services and activities

Improved mental wellbeing.

Improved quality of life

Improved digital skills.

**System benefits:**

Improved understanding of barriers to participation in exercise programmes by older people.

Workforce upskilled.

Evidence led commissioning.

**Assumptions**

Effectiveness

- Participants are at high risk of falling and inactive at the start
- Participants make expected physical and psychological improvements
- Physical improvements lead to a reduction in falls

Fidelity

- PSIs adhere to the FaME programme, with progressively difficult exercises
- Participants adhere to the FaME programme (continue for 6 months, do home exercises)
- Commissioners commission programmes with adequate dose, frequency and duration

Barriers/facilitators

- There is a pool of level 3 instructors available to train
- The trained PSIs stay local/continue to be available to public sector
- People want to exercise
- People want to exercise in groups
- Referrers know about FaME
- Referrers believe FaME is effective
- People can get to the courses (transport, venue locations, cost)
- Suitable venues are available to deliver FaME in
- Instructors have time to talk to participants, building a good relationship
- People get on with each other during groups/there are opportunities to bond as a group
