# Supplementary material for: A mixed-methods formative process evaluation of the falls management exercise programme in an English county
Source: BMC Public Health. 2025 Aug 1;25:2609. doi: 10.1186/s12889-025-23737-6 (PMC12315209; doi:10.1186/s12889-025-23737-6)
Supplement: Supplementary file 2 — Supplementary Material 2. [file 12889_2025_23737_MOESM2_ESM.docx]

Details of Ordinal Scales used for the Timed-up-and-go, Turn 180^0^, and Functional Reach tests

| **Timed-up-and-go** | Able to rise from the chair easily, walk unaided, turn without dizziness or stumbling. Faster than 8 seconds | Able to rise from the chair without arms, walk unaided, turn without stumbling. Between 9 and 15 seconds | Difficulty on rising from chair, walks aided or unaided but takes between 16 and 24 seconds | Difficulty on rising from chair, walks aided or unaided but takes between 25 and 40 seconds | Difficulty on rising from chair, needs walking aid, unconfident or dizzy on turning, takes over 40 seconds | Unable to rise from the chair, walk or tuns without help |
| --- | --- | --- | --- | --- | --- | --- |
|  | 1 | 2 | 3 | 4 | 5 | 6 |
| **Turn 180^0^** | Able to turn safely in 4 steps or less | Able to turn safely in 6 steps or less | Able to turn safely in 8 steps or less | Needs close supervision and verbal cueing for safety. Takes 9-12 steps | Needs assistance from one person or furniture to turn | Needs assistance from 2 people to turn |
|  | 1 | 2 | 3 | 4 | 5 | 6 |
| **Functional reach** | Reaches greater than 35 cm | Reaches between 25 and 34 cm | Reaches between 16 and 24 cm | Reaches between 10 and 15 cm | Reaches less than 10cm | Unable or afraid to reach forward |
|  | 1 | 2 | 3 | 4 | 5 | 6 |
