# Supplementary material for: A mixed-methods formative process evaluation of the falls management exercise programme in an English county
Source: BMC Public Health. 2025 Aug 1;25:2609. doi: 10.1186/s12889-025-23737-6 (PMC12315209; doi:10.1186/s12889-025-23737-6)
Supplement: Supplementary file 3 — Supplementary Material 3. [file 12889_2025_23737_MOESM3_ESM.docx]

**Formative process evaluation of the Falls Management Exercise (FaME) Programme in Lincolnshire**

**Topic Guide: Interviews with Commissioners and managers**

*Note: This topic guide is indicative. It is a guide to the topics to be covered during the interviews (and not a script) therefore the order of topics, and the precise manner in which they are addressed, will be flexible according to the circumstances.*

*The topics will be covered across interviews 1 and 2. It is not anticipated that all topics will be addressed in every interview.*

**Introduction:**

Introduce the study

Talk through key points:

- length of interview
- like a discussion, but will cover key topics
- no right or wrong answers
- participation is voluntary, rights to withdraw
- recording; audio only
- confidentiality and anonymity
- consent (and form)

**Background Information:**

- Role and responsibilities
- Role and responsibilities in relation to FaME
- Background to using FaME
  - Falls amongst older adults in Lincolnshire – identifying the problem
  - Other/previous services for this group
  - Reasons for implementing FaME
- Relationship between Lincolnshire County Council and One You Lincolnshire
- How is FaME being delivered in Lincolnshire? (locations, numbers on programme)

**Implementation set-up:**

- Use of guidance such as the commissioning toolkit and related materials
  - Usefulness, any adaptations
- Recruiting PSIs
  - Who has been recruited
  - Challenges with recruitment/retention
- Training PSI instructors
  - How, by whom
  - Ongoing support provided
  - Successes/challenges of this
- Identifying FaME class venues
  - How is this done
  - Suitability of venues
  - Challenges/advantages in Lincolnshire

**Referrals:**

- How are participants identified?
  - PHM dataset
  - Clinician identified
- How are participants informed about FaME?
- Who refers to the programme? How is this managed?
- Appropriateness of referrals
- Recruitment rates (and challenges)
- Participation/uptake
- Participant retention (and reasons for dropout)
- What is working well/not working?
- Any planned changes?

**Participant response:**

- How do participants react to being referred to FaME?
- What do they think of the programme?
- How do they respond to the classes?
- Does this differ across locations/instructors?
- Do participants progress with the exercises? (differences; strategies to encourage progression)
- How well do participants engage with the PSIs?
- Do participants participate in at-home exercise?
  - What helps with this/challenges
- What is in place to support physical activity after the FaME programme?
  - Signposting and uptake of services
  - Other PA services
  - Successes/challenges

**Participant impact**

- How well is outcome monitoring being managed?
- Issues with data collection
- Participant feedback
- Participant outcomes
  - Strength and balance measures
  - Falls
  - Physical activity
  - Quality of life
  - Other impacts

**Implementation fidelity:**

- Is the FaME programme being delivered as intended?
  - Use of implementation toolkit
  - Key elements of FaME
  - Differentiation across delivery venues/instructors
  - Challenges (and in Lincolnshire)
  - Changes/adaptations and why
- How well did collecting the routine data from participants work?
- What other quality assurance was in place? Success of this?
- Review of logic model

**Lessons for future roll-out**

- Main challenges/successes in Lincolnshire
- What will you change? And why?
- Lessons for other areas implementing FaME
