# Supplementary material for: A mixed-methods formative process evaluation of the falls management exercise programme in an English county
Source: BMC Public Health. 2025 Aug 1;25:2609. doi: 10.1186/s12889-025-23737-6 (PMC12315209; doi:10.1186/s12889-025-23737-6)
Supplement: Supplementary file 4 — Supplementary Material 4. [file 12889_2025_23737_MOESM4_ESM.docx]

**Formative process evaluation of the Falls Management Exercise (FaME) Programme in Lincolnshire**

**Topic Guide: Interviews with Participants (and carers)**

*Note: This topic guide is indicative. It is a guide to the topics to be covered during the interviews (and not a script) therefore the order of topics, and the precise manner in which they are addressed, will be flexible according to the circumstances.*

**Introduction:**

Introduce the study

Talk through key points:

- length of interview
- like a discussion, but will cover key topics
- no right or wrong answers
- participation is voluntary, rights to withdraw
- recording; audio only
- confidentiality and anonymity, data protection
- consent (and form)

**Background Information:**

- Tell me about yourself – introductory chat
- **START RECORDING**
- Level of physical activity
  - Changes in last 6 months
- History of falls
  - Impact/consequence
- Confidence/fear of falls

**Learning about FaME:**

- How did you first hear about FaME?
  - Who/when
- How much information did you have about FaME?
  - Format
  - Enough to make a decision?
- Initial thoughts on attending
- Reasons for attending (or not)
- Hopes and aspirations of programme
- Any conversation with family/carers about attending?
- How long was it after hearing about it did you attend the first class?

**Practicalities:**

- How do you travel to the FaME class?
- What time of the week is it?
  - Advantages/disadvantages of this
- Is there anything that makes it difficult to attend? Why?
- Have you missed any classes?
  - Why and impact of this
- What do you think about the venue?

**Experience of FaME sessions:**

- Please tell me what the FaME classes are like.
- How do you find the exercises?
- Are any of them difficult?
- Do they get easier/more difficult each week?
- Do you find them helpful?
- What are the other participants like?
  - Have you made friends
- What is the PSI like?
  - Are they welcoming?
  - Do they explain the exercises clearly>
  - Do you feel supported?
- Would you change anything about the class?
- Do you stay for tea/chat after?
  - Do you enjoy this?
- Do you exercise at home/between classes
  - What do you do
  - Use of any booklet/online guide
  - Is this useful
  - If you don’t exercise between classes, why is this?
- What do you particularly like about FaME?
- And what do you dislike/would prefer was done differently.

**(for those who dropped out)**

- Why did you stop attending?
- Could anything have been done to prevent this?
- Would you like to try FaME again in the future?

**Impact**

- What do you think have been the benefits of FaME for you
- Has it changed your
  - Worry about falls
  - Level of physical activity
  - Confidence getting out and about
  - Quality of life
  - Friendships
  - Relationships with family/carers
  - Impact on carers/family
  - Staying independent
  - Other impacts (digital skills)
- What is it about FaME that made this happen?
- (if post-FaME) do you do any activities that you didn’t do before?
- Were you told about opportunities to attend any other groups or activities to help you stay active
  - Attendance are reasons
- Is there anything else you would like to tell me about FaME?
