# Supplementary material for: A mixed-methods formative process evaluation of the falls management exercise programme in an English county
Source: BMC Public Health. 2025 Aug 1;25:2609. doi: 10.1186/s12889-025-23737-6 (PMC12315209; doi:10.1186/s12889-025-23737-6)
Supplement: Supplementary file 5 — Supplementary Material 5. [file 12889_2025_23737_MOESM5_ESM.docx]

**Formative process evaluation of the Falls Management Exercise (FaME) Programme in Lincolnshire**

**Topic Guide: Interviews with PSIs**

*Note: This topic guide is indicative. It is a guide to the topics to be covered during the interviews (and not a script) therefore the order of topics, and the precise manner in which they are addressed, will be flexible according to the circumstances.*

*The topics will be covered across interviews 1 and 2. It is not anticipated that all topics will be addressed in every interview.*

**Introduction:**

Introduce the study

Talk through key points:

- length of interview
- like a discussion, but will cover key topics
- no right or wrong answers
- participation is voluntary, rights to withdraw
- recording; audio only
- confidentiality and anonymity
- consent (and form)

**Background Information:**

- Professional background
- Role and responsibilities
- Role and responsibilities in relation to FaME
  - Number of locations/classes
- How is FaME being delivered in Lincolnshire? (locations, numbers on programme)

**Implementation set-up:**

- Use of guidance such as the commissioning toolkit and related materials
  - Usefulness, any adaptations
- Training PSI instructors
  - How, by whom
  - Ongoing support provided
  - Successes/challenges of this
- Identifying FaME class venues
  - How is this done
  - Suitability of venues
  - Challenges/advantages in Lincolnshire

**Referrals:**

- Appropriateness of referrals
- Recruitment rates (and challenges)
- Participation/uptake
- Participant retention (and reasons for dropout)
- What is working well/not working?
- Any planned changes?

**Participant response:**

- How do participants react to being referred to FaME?
- What do they think of the programme?
- How do they respond to the classes?
- Do participants progress with the exercises? (differences; strategies to encourage progression)
- How well do participants engage with the you?
- Do participants participate in at-home exercise?
  - What helps with this/challenges
- What is in place to support physical activity after the FaME programme?
  - Signposting and uptake of services
  - Other PA services
  - Successes/challenges
- Do participants stay for the social chat after classes?
  - Importance of this
  - Interactions between participants/carers/PSI at this time
  - What does this add to the programme?

**Participant impact**

- Participant feedback
- Carer feedback
- Participant outcomes
  - Strength and balance measures
  - Falls
  - Physical activity
  - Quality of life
  - Other impacts

**Implementation fidelity:**

- What do you think of the FaME programme?
- What is it like to deliver?
  - Challenges
  - Optimal number in class?
- Would you change anything about the programme?
- Is the FaME programme being delivered as intended?
  - Use of implementation toolkit
  - Key elements of FaME
  - Challenges (and in Lincolnshire)
  - Changes/adaptations and why
- Review of logic model

**Lessons for future roll-out**

- Main challenges/successes in Lincolnshire
- What would you change? And why?
- Lessons for other PSIs implementing FaME
